# The morphological features of different Schatzker types of tibial plateau fractures: a three-dimensional computed tomography study

**DOI:** 10.1186/s13018-016-0427-5

**Published:** 2016-08-27

**Authors:** Pengbo Chen, Hao Shen, Wei Wang, Binbin Ni, Zhiyuan Fan, Hua Lu

**Affiliations:** Department of Orthopaedics, Xinhua Hospital Affiliated to Shanghai Jiaotong University School of Medicine, No. 1665 Kongjiang RD, Shanghai, 200092 China

**Keywords:** Tibial plateau fracture, Schatzker type, Morphological feature, Computed tomography

## Abstract

**Background:**

Tibial plateau fractures are of great challenge to treat with open reduction and internal fixation, because fractures vary from simple to complex, with little or extensive articular involvement. Hence, recognition and comprehension of the fracture features will help orthopedic surgeons understand the injury mechanism better and manage these fractures by planning optimal surgical procedures. This study aimed to evaluate the morphological characteristics of tibial plateau fractures based on the Schatzker classification.

**Methods:**

A total of 186 patients with 188 tibial plateau fractures from 2010 to 2014 in our hospital were reviewed using a computed tomography scan and three-dimensional (3D) reconstruction. The main fracture line angles (FLA) of Schatzker types I, II, and IV were measured. For each fracture, depression depth was measured, and the depression zone was also located. Depression zones were overlapped to obtain a frequency diagram.

**Results:**

Schatzker type I and II fractures had three subtypes: single anterolateral fracture, single posterolateral fracture, and complex fracture (the anterolateral and posterolateral parts). Schatzker type IV fractures were also divided into three subtypes: single posteromedial fracture, single anteromedial fracture, and the whole medial fracture. For various Schatzker types and subtypes of fracture, fracture depression clustered and occurred at different locations of the tibial plateau. A significant difference was observed in the depression depth among the different Schatzker types (*P* < 0.01, Kruskal-Wallis test), especially between Schatzker type III and other types (Nemenyi test). There was no difference in the depression depth among the subtypes of Schatzker type II, whereas the difference was significant between the two subtypes of Schatzker type IV.

**Conclusions:**

Schatzker type I, II, and IV fractures could be divided into three corresponding subtypes by FLA. Various Schatzker types of fractures differed in location and depth of depression. A proper operative approach should be made based on the morphological characteristics of individual types of tibial plateau fractures.

## Background

Tibial plateau fractures, one intra-articular fracture caused by high-velocity trauma, are of great challenge to treat [1, 2]. Treatment with open reduction and internal fixation is difficult [2–4] because fractures vary from simple to complex, with little or extensive articular involvement [5, 6]. Hence, recognition and comprehension of the fracture features will help orthopedic surgeons understand the injury mechanism better and manage these fractures by planning optimal surgical procedures. Now, three classification systems have been commonly used for classifying tibial plateau fractures in clinical practice, bicondylar including Schatzker, AO/OTA, and Three-Column classifications [7–9]. Based on X-ray plain radiographs, Schatzker and AO classifications describe the location and general pattern of fracture without consideration of the fracture line orientation, which usually determines the right position of the bone plate. They were also lacking in adequate details of depression morphological characteristics. However, identification of depression morphological characterization would facilitate surgical plan and therapeutic effect postoperatively [10]. Failure to accomplish the reduction of depression is associated with residual pain, post-traumatic arthritis, and deformity [10–12].

Computed tomography (CT) is nowadays indispensable in the understanding of fracture patterns precisely, especially in consideration of fracture line orientation, location, and magnitude of depression components [13, 14]. It provides surgeons with an opportunity to promote the ability of reduction and internal fixation. Although the Three-Column Classification system locates the fracture based on CT [8], it overlooks the fracture line orientation and morphological characteristics of depression.

Hence, this study was to clarify the morphologic characteristics of different Schatzker types [7] of tibial plateau fractures based on CT scanning and three-dimensional (3D) reconstruction, by measuring the fracture line angles (FLA), mapping the most common zones of depression, and measuring the depression depth. Our study would give the surgeons instructions during diagnosis, preoperative planning, and execution of surgical strategies.

## Methods

### Patients

The retrospective study reviewed a total 186 patients with tibial plateau fractures who were treated in our hospital between January 2010 and December 2014. For each patient, computed tomography (CT) scans of the knee have been performed. Exclusion criteria were age of less than 16 years old, incomplete CT images, previous knee surgery, and/or existing knee deformity. Additionally, any patient with an open proximal tibial fracture was excluded. Demographic and clinical data such as age, gender, causes of fracture, affected side, Schatzker type, and accompanied injury were collected from the medical records department.

### 3D reconstruction

CT images were saved in an interactive medical image processing software (Mimics 16.0, Materialise, Leuven, Belgium) [15], which allowed identifying the issues with different CT values visible on the scans. Mimics featured extended visualization and segmentation functions based on image density thresholding. We selected the CT data of bone according to the “Thresholding” (CT value > 225) command on the entire stack of scans, and built the mask of tibia by the “Region Growing” command, followed by noise filtering. Then, the 3D analogous image of each fracture was generated.

### FLA

**Fig. 1:**
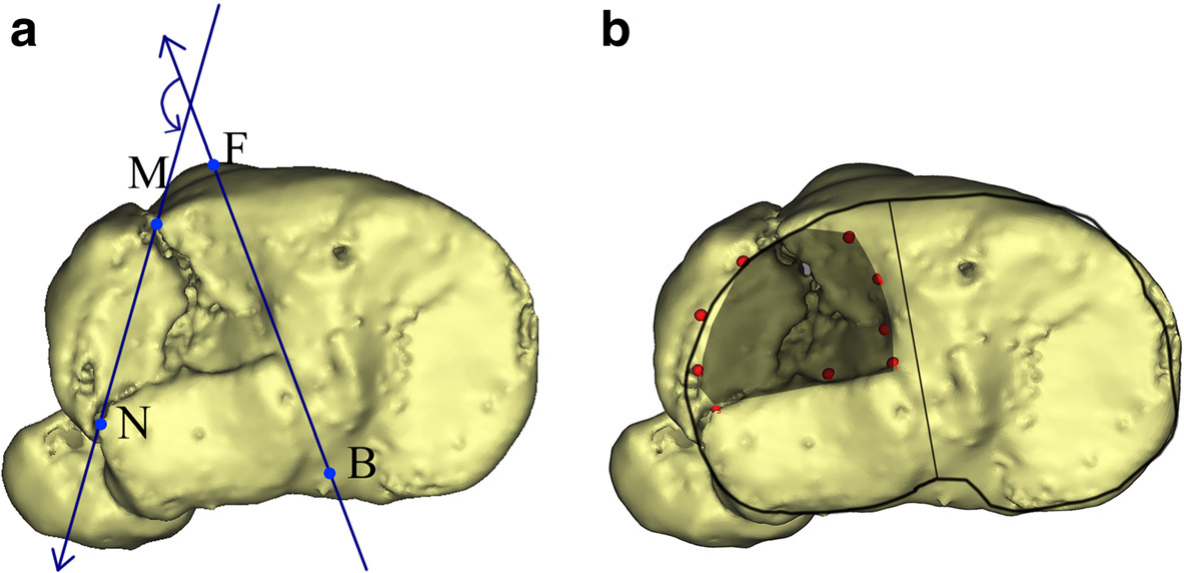
The method to calculate the fracture line angle (FLA) and depict the depression. **a** The FLA is determined by measuring the included angle between *BF* and major fracture line *MN. B* is the middle point of the posterior cruciate ligament’s insertion on the tibial plateau; *F* is the medial 1/3 point of the tibial tuberosity. *BF* is used as the reference line to determine the major FLA. **b** The depression zone is depicted with a certain gray value and transparency level. The *red points* at the border are the position with ≥2 mm depression, but not all the borders are of less than 2 mm of depression

In the top view, the line connecting the middle point of the posterior cruciate ligament’s insertion (B) on the tibial plateau and the medial 1/3 point of the tibial tuberosity (F) acted as a neutral axis. The FLA was determined by counterclockwise measuring the included angle between BF and major fracture line MN of the separated fragment when the fractures occurred in the left leg (Fig. 1a); otherwise, the clockwise FLA was determined. Considering that Schatzker type V and VI fractures were too complex to be measured accurately, only the FLA of Schatzker type I, II, and IV fractures were measured.

### Mapping of fracture depression zone

As all tibial plateau fractures may combine with more or less depression, a 2-mm depression was considered as a threshold for depression mapping and depth measurement in this study. The method of mapping the fracture line or depression for a given bone, as Cole and colleagues previously described [16, 17], was used for this study. After 3D reconstruction, the border of the depression zone was marked on the top view image. Then, the fracture image was imported into Macromedia Fireworks (v7.0, Macromedia Inc., San Francisco, California) and superimposed to a normal tibial plateau image according to the posterior margin and the line BF, with the size adjusted properly to match the normal. According to the marked border on the fracture image, the depression area was depicted on the normal image with a certain gray value and transparency level (Fig. 1b). Every depression zone was depicted on this normal image as well. With the fracture images superimposed, the gray value of the overlapped area increased. The final gray value (frequency diagram) indicated the incidence of depression at the certain location.

### Depression depth

**Fig. 2:**
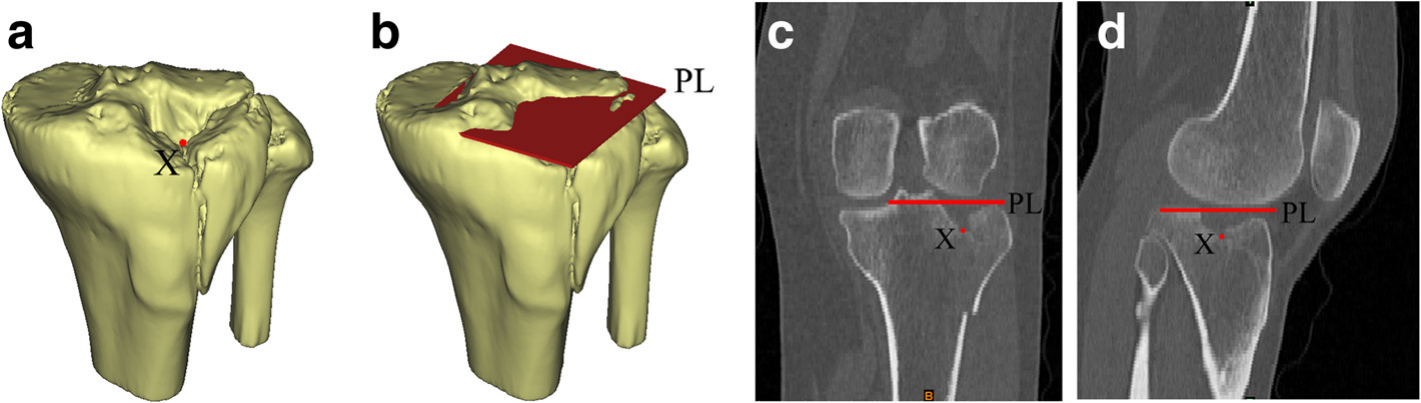
The method to calculate the depression depth. **a** *X* is the most depressed point of the articular surface. **b** *PL* is the normal articular surface plane. **c** Anteroposterior. **d** Lateral. The depression depth is obtained by calculating the distance from *X* to *PL*

Using Mimics software (v16.0, Materialise, Leuven, Belgium), the depression depth was measured. The most depressed point X of the articular surface (Fig. 2a), as well as the normal articular surface PL (Fig. 2b), was marked for each fracture after the accurate evaluation by CT scans and the 3D models. Then, the depression depth was obtained by automatically calculating the distance from X to PL (Fig. 2c, d) with Mimics software.

### Statistical analyses

Qualitative data are shown as *n* (percentage) and quantitative data are expressed as mean ± SD. The depression depths among different Schatzker types and subtypes were compared using Kruskal-Wallis and Nemenyi tests. All statistical analyses were performed using SAS V8 software (SAS Institute Inc., North Carolina, USA). Difference was considered significant if the *P* value was less than 0.05.

## Results

Patient demographic and clinical data are summarized in Table 1. In our study, there were 106 male and 80 female patients, with an average age of 54.9 years (range 17–87 years) at the time of injury. Two of them had bilateral tibial plateau fractures. A total of 188 CT scans were reviewed: 115 showed Schatzker type I (12), II (74), or III (29) tibial plateau fractures; 15 showed a Schatzker type IV tibial plateau fracture; and 58 showed Schatzker type V (30) or type VI (28) tibial plateau fractures. There were 159 (84.6 %) tibial plateau fractures with depression.

**Table 1:** Demographic and clinical data of patients with tibial plateau fractures

Parameter	Patients
Mean age (range), years	54.9 (17–87)
Gender (male/female), *n*	106/80
Causes of fracture, *n* (%)	
Road traffic accidents	79 (42.5 %)
Falling from a height	45 (24.2 %)
Riding electromobile injuries	38 (20.4 %)
Others	24 (12.9 %)
Affected side, *n* (%)	
Left	112 (60.2 %)
Right	72 (38.7 %)
Bilateral	2 (1.1 %)
Simple fracture, *n* (%)	130 (69.9 %)
Complicated fracture, *n* (%)	56 (30.1 %)
Schatzker classification (*N* = 188)	
Type I	12
Type II	74
Type III	29
Type IV	15
Type V	30
Type VI	28
Associated injury, *n*	
Other fracture within the knee joint	45
Non-knee joint fracture	38
Organic lesion	32
Craniocerebral injury	23

The FLA of Schatzker type I and II fractures ranged from 90° to 270°, whereas the Schatzker type IV fracture distributed between 270° and 450°. Mean FLA of Schatzker type I, II, and IV tibial plateau fractures is summarized in Table 2. On the basis of the FLA, both Schatzker type I and II fractures were further categorized into three subtypes: single anterolateral fracture (SALF), single posterolateral fracture (SPLF), and complex fracture (CF), including anterolateral and posterolateral parts. The average FLA of these fractures were 145.92° ± 16.06°, 208.87° ± 6.85°, 149.59° ± 9.33° and 248.80° ± 11.53° (Schatzker type I) and 149.66° ± 13.17°, 235.79° ± 19.85°, 127.41° ± 16.51° and 231.62° ± 14.71° (Schatzker type II), respectively. The Schatzker type IV fracture was also divided into three subtypes: single posteromedial fracture (SPMF), single anteromedial fracture (SAMF), and whole medial fracture (WMF), with the average FLA of 293.27° ± 26.76°, 409.66° ± 17.24°, and 341.10° ± 16.34°, respectively.

**Table 2:** Fracture line angle (FLA) of different Schatzker types of tibial plateau fractures

Schatzker type	FLA (°)
Type I	
Single anterolateral fracture (SALF)	145.92 ± 16.06
Single posterolateral fracture (SPLF)	208.87 ± 6.85
Anterolateral part of complex fracture (CF)	149.59 ± 9.33
Posterolateral part of CF	248.80 ± 11.53
Type II	
SALF	149.66 ± 13.17
SPLF	235.79 ± 19.85
Anterolateral part of CF	127.41 ± 16.51
Posterolateral part of CF	231.62 ± 14.71
Type IV	
Single posteromedial fracture (SPMF)	293.27 ± 26.76
Single anteromedial fracture (SAMF)	409.66 ± 17.24
Whole medial fracture (WMF)	341.10 ± 16.34

**Fig. 3:**
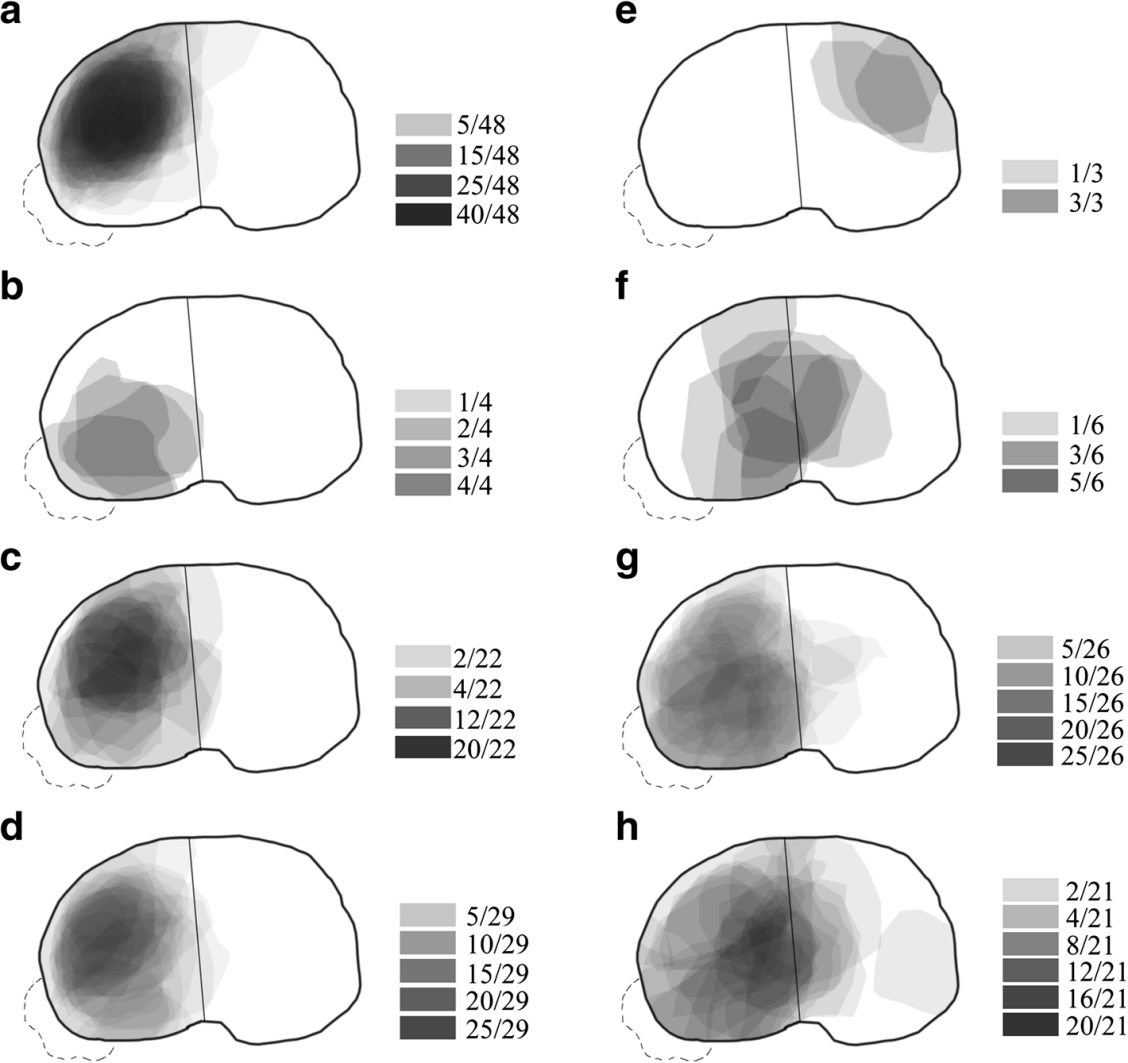
The “heat maps” of tibial plateau fractures with depression zone. **a**–**c** single anterolateral fracture (SALF), single posterolateral fracture (SPLF), and complex fracture (CF) of Schatzker type II. **d** Schatzker Type III. **e**–**f** Single anteromedial fracture (SAMF) and whole medial fracture (WMF) of Schatzker type IV. **g** Schatzker type V. **h** Schatzker type VI. The final gray value (frequency diagram) indicated the incidence of depression at the certain location

Mapping of the 159 tibial plateau fractures resulted in a diverse diagram of fracture zones of depression (Fig. 3). The complete fracture map was divided according to fracture types for visual comparison and analysis. From Schatzker types II to VI, the “heat maps” of depression were depicted. The Schatzker type II fracture had three subtypes according to the FLA, leading to three predictable pattern “heat maps” (Fig. 3a–c), as the tibial plateau depressed surrounding the fracture lines. The depression zone of Schatzker type III is located at the anterolateral tibial plateau (Fig. 3d). Despite the three subtypes of the Schatzker type IV fracture, only two “heat maps” were depicted because SPMF patients had no depression in our cohort (Fig. 3e, f). Beyond our expectations, WMF depressed more frequently at the center and posterolateral side of the tibial plateau. The depression zones of Schatzker type V and VI fractures distributed at the whole lateral plateau (Fig. 3g, h). However, the Schatzker type V fracture was most likely to cause depression at the posterolateral side whereas the Schatzker type VI fracture at the center of the tibial plateau.

Comparisons of the depression depths between different types are shown in Table 3. There was a significant difference in depression depth among different Schatzker type fractures by Kruskal-Wallis test (*P* < 0.01). The severity order of the average depression depth and mean Wilcoxon score were Schatzker types III, II, V, VI, and IV (Table 3). Furthermore, the analysis by Nemenyi test also confirmed a less depression depth of the Schatzker type III fracture than the other types of fractures (Table 3). Further analyses revealed a similar depression depth among the three subtypes of the Schatzker type II fracture (*P* = 0.2374, Table 3); however, as for the Schatzker type IV fracture, the difference was significant between the SAMF and WMF subtypes (*P* = 0.0404).

**Table 3:** Depression depth of different Schatzker types and subtypes of tibial plateau fractures

Schatzker type	Depression rate (%)	Depression depth, mm	Mean score
Type II	74 (100 %)	7.85 ± 4.24 a	73.43
Single anterolateral fracture (SALF)	48 (64.9 %)	8.43 ± 4.26	–
Single posterolateral fracture (SPLF)	4 (5.4 %)	5.59 ± 2.74	–
Complex fracture (CF)	22 (29.7 %)	7.13 ± 4.35	–
Type III	29 (100 %)	5.16 ± 1.49 b	44.71
Type IV	9 (60.00 %)	16.7 ± 8.84 a	120.44
Single posteromedial fracture (SPMF)	0	–	–
Single anteromedial fracture (SAMF)	3 (33.3 %)	8.15 ± 3.40	–
Whole medial fracture (WMF)	6 (66.7 %)	17.93 ± 10.05	–
Type V	26 (86.67 %)	11.99 ± 6.93 a	101.81
Type VI	21 (75.00 %)	13.25 ± 8.73 a	107.57

Fracture types with different letters means that depression depth were significant different between different Schatzker types

## Discussion

Tibial plateau fracture requires great and precise understanding of fracture features for optimal preoperative planning. On the base of radiographic images, Schatzker and AO/OTA classifications describe the location and general pattern of fracture line but without considering the orientation of fracture line [7, 9]. CT and 3D reconstruction software have been recognized as important tools in preoperative protocol to understand radiographic images [8, 18]. Despite the use of CT scanning to assess the location of fracture lines [8], the Three-Column Classification also lacks the discrimination of fracture line orientation. Meanwhile, none of the classifications aforementioned has taken into account the morphological characteristics of depression. Fracture line location is of great importance for the planning of an appropriate surgical approach [19–21]. Depression is the other factor that will influence the choice of surgical approach, anatomical reduction or functional restoration, and even the development of periarticular plates [22]. After the analyses of CT images and 3D reconstruction from 188 different Schatzker types of tibial plateau fractures, this study revealed the distribution of fracture line orientation by the FLA and exhibited the associated morphological characteristics of depression by “heat maps” and depth.

Schatzker type I, II and III fracture occur at the lateral tibial plateau. Both Schatzker type I and II fractures had three subtypes (SALF, SPLF, and CF). The SALF can be exposed well by the anterolateral approach according to the statistics of the FLA. However, it was still controversial to expose and treat the posterolateral fracture parts of both SPLF and CF; posterolateral and anterolateral approaches with or without fibula osteotomy were suggested [23–25]. Frosch et al. proposed a posterolateral skin incision with two approaches, which allowed the exposure and examination of the posterolateral joint surfaces of the tibia [24]. Solomon et al. described a posterolateral trans-fibular neck approach to the proximal tibia, which needed an osteotomy and fixation [25]. Meantime, Yu et al. suggested partial fibular head osteotomy [23]. According the average FLA of SPLF, the fibular head will be a great obstacle for exposure and reduction. Screws are most efficient when placed perpendicular to the fracture plane [26]. Consequently, the posterolateral approach with fibula osteotomy could be an efficient and direct way to achieve the goal of anatomical reduction and stable fixation. As for the CF, anterolateral and posterolateral fractures might have a better outcome when treated by bilateral-approach and dual-plate corresponding according to the FLA. Sun and his colleagues also approved of this surgical treatment after clinical observations [27]. Besides, a proper operative approach would make for the stabilizing pattern of fracture reduction. Furthermore, a systemic understanding of fracture morphology such as the FLA would make the inserter internal fixities (hollow screw, blade plate, or intramedullary pin) designed more specially for individual patient, which will also be conducive to the postoperative fracture stability.

In this study, SALF and CF of Schatzker type II were both more likely to depress at the anterolateral side of the tibial plateau, so with Schatzker type III. The larger depth of Schatzker type II compared with Schatzker type III might suggest that the Schatzker type II fracture was caused by a more powerful violence. The SPLF of Schatzker type II had an associated depression area with the fracture line. As the depression depths of the three subtypes of the Schatzker type II fracture were not significantly different, it was presumed that the violence might be also similar.

The WMF of the Schatzker type IV fracture was shown with a depression at the center and posterolateral side of the tibial plateau. The violence of WMF might be greater than that of the SAMF according to the depression depth (*P* < 0.01). Sufficient care should be taken for the depression of it [28]. The mean WMF line angle was 341.10° ± 16.34°, suggesting that the fracture lines went more frequently from anteromedial to posterolateral. Considering this, a posteromedial approach or a medial approach was needed for this subtype of the Schatzker type IV fracture. SAMF and SPMF with or without depression surrounding the fracture line accounted for a low proportion of fractures. The SAMF with an average FLA of 409.66° ± 17.24° would be well-exposed and fixed through an anteromedial approach, whereas SPMF with a FLA of 293.27° ± 26.76° needed to be treated via a posteromedial approach [22, 29]. The “heat maps” of Schatzker type V and VI fractures revealed that the anatomical reduction or functional restoration for depression of them were difficult as the depression was located more frequently in the center and the posterolateral side of the tibial plateau. However, not all tibial plateau fractures require open reduction, and fractures of less than 2 mm of depression are stabilized with casting, pinning, or subcutaneously plating without fragment reduction. Rasmussen also advised that an operation should be performed when the depression depth arrives at 5 mm [30]. Therefore, distinguishing the depression with 2 mm for fractures is critical.

Apart from the morphological features of different Schatzker types of tibial plateau fractures, we further summarized two key methods of measurement in this study, one for measuring FLA and the other for depression zone and depression depth. Although the FLA was measured only for Schatzker type I, II, and IV fractures because of the difficulty in identifying the anatomical biomarker in Schatzker type V and VI fractures, this can be regularly applied to all preoperative cases as long as the anatomical biomarkers were recognized. When a patient was encountered with a tibial plateau fracture in clinic, Schatzker type was first determined for preliminary judgment, and then the FLA was detected using the method as described in this study, followed by the determination of fracture subtype. Thereby, special management for this subtype of tibial plateau fracture could be performed. For example, if a patient was classified as a Schatzker type IV as well as WMF subtype after the measurement of FLA, the actual depression area and depth of the patient could be determined, as this subtype has been proved with depression at the center and posterolateral areas of the tibial plateau. Accordingly, based on comprehensive, complete, and clear understanding, a posteromedial approach was preferred with a specially designed blade plate that was conducive to the insertion of the screw perpendicular to the major fracture line, so was the case with other Schatzker types of tibial plateau fractures. Hence, this study, to a certain extent, would provide guidance or some experiences to the surgeons during diagnosis, preoperative planning, and execution of surgical strategies.

It was a limitation of this study that the FLA of Schatzker type V and VI fractures was not measured due to their complexity. The other limitation was the small sample size, especially the number of Schatzker type IV fractures, which may lead to statistics deviation when compared with the total population of tibial plateau fractures.

## Conclusions

The data and “heat maps” here elucidated the patterns of the tibia plateau fractures: Schatzker type I, II, and IV fractures could be divided into three corresponding subtypes by FLA. The location and depression depth of different Schatzker types of fractures were highly variable. Proper preoperative planning, operative approach, and fixation method should be selected based on the morphological characteristics of individual types of plateau fractures.

## Abbreviations

3D: Three-dimensional
ANOVA: Analysis of variance
CF: Complex fracture
CT: Computed tomography
FLA: Fracture line angles
SALF: Single anterolateral fracture
SAMF: Single anteromedial fracture
SPLF: Single posterolateral fracture
SPMF: Single posteromedial fracture
WMF: Whole medial fracture

## References

[1] Lowe JA, Tejwani N, Yoo B, Wolinsky P (2011). Surgical techniques for complex proximal tibial fractures. J Bone Joint Surg Am.

[2] Hall JA, Beuerlein MJ, McKee MD. Open reduction and internal fixation compared with circular fixator application for bicondylar tibial plateau fractures. J Bone Joint Surg Am. 2009;91 Supp 2 (Part 1):74-88. 10.2106/JBJS.G.01165 19255201

[3] Johnson EE, Timon S, Osuji C (2013). Surgical technique: Tscherne-Johnson extensile approach for tibial plateau fractures. Clin Orthop Relat Res.

[4] Berkson EM, Virkus WW (2006). High-energy tibial plateau fractures. J Am Acad Orthop Surg.

[5] Barei DP, O’Mara TJ, Taitsman LA, Dunbar RP, Nork SE (2008). Frequency and fracture morphology of the posteromedial fragment in bicondylar tibial plateau fracture patterns. J Orthop Trauma.

[6] Streubel PN, Glasgow D, Wong A, Barei DP, Ricci WM, Gardner MJ (2011). Sagittal plane deformity in bicondylar tibial plateau fractures. J Orthop Trauma.

[7] Schatzker J, Mcbroom R, Bruce D (1979). The tibial plateau fracture: the Toronto experience 1968-1975. Clin Orthop Relat Res.

[8] Zhu Y, Yang G, Luo CF, Smith WR, Hu CF, Gao H (2012). Computed tomography-based Three-Column Classification in tibial plateau fractures: introduction of its utility and assessment of its reproducibility. J Trauma Acute Care Surg.

[9] Müller ME, Nazarian S, Koch P, Schatzker J. The comprehensive classification of fractures of long bones. Berlin, Heidelberg: Springer Science & Business Media; 2012.

[10] Tscherne H, Lobenhoffer P (1993). Tibial plateau fractures: management and expected results. Clin Orthop Relat Res.

[11] Onderko LL, Rehman S (2013). Treatment of articular fractures with continuous passive motion. Orthop Clin North Am.

[12] Urruela AM, Davidovitch R, Karia R, Khurana S, Egol KA (2013). Results following operative treatment of tibial plateau fractures. J Knee Surg.

[13] Atesok K, Finkelstein J, Khoury A, Peyser A, Weil Y, Liebergall M (2007). The use of intraoperative three-dimensional imaging (ISO-C-3D) in fixation of intraarticular fractures. Injury.

[14] Gösling T, Klingler K, Geerling J, Shin H, Fehr M, Krettek C (2009). Improved intra-operative reduction control using a three-dimensional mobile image intensifier—a proximal tibia cadaver study. Knee.

[15] Magne P, Stanley K, Schlichting LH (2012). Modeling of ultrathin occlusal veneers. Dent Mater.

[16] Cole PA, Mehrle RK, Bhandari M, Zlowodzki M (2013). The pilon map: fracture lines and comminution zones in OTA/AO type 43C3 pilon fractures. J Orthop Trauma.

[17] Armitage BM, Wijdicks CA, Tarkin IS, Schroder LK, Marek DJ, Zlowodzki M (2009). Mapping of scapular fractures with three-dimensional computed tomography. J Bone Joint Surg Am.

[18] Suero EM, Hüfner T, Stübig T, Krettek C, Citak M (2010). Use of a virtual 3D software for planning of tibial plateau fracture reconstruction. Injury.

[19] Berber R, Lewis CP, Copas D, Forward DP, Moran CG (2014). Postero-medial approach for complex tibial plateau injuries with a postero-medial or postero-lateral shear fragment. Injury.

[20] Tornetta P, Weiner L, Bergman M, Watnik N, Steuer J, Kelley M (1993). Pilon fractures: treatment with combined internal and external fixation. J Orthop Trauma.

[21] Yang G, Zhu Y, Luo CF, Putnis S (2012). Morphological characteristics of Schatzker type IV tibial plateau fractures: a computer tomography based study. Int Orthop.

[22] Hahnhaussen J, Hak DJ, Weckbach S, Heiney JP, Stahel PF (2012). Percutaneous inflation osteoplasty for indirect reduction of depressed tibial plateau fractures. Orthopedics.

[23] Yu BQ, Han KW, Zhan C, Zhang CC, Ma H, Su JC (2010). Fibular head osteotomy: a new approach for the treatment of lateral or posterolateral tibial plateau fractures. Knee.

[24] Frosch KH, Balcarek P, Walde T, Stürmer KM (2010). A new posterolateral approach without fibula osteotomy for the treatment of tibial plateau fractures. J Orthop Trauma.

[25] Solomon LB, Stevenson AW, Baird RP, Pohl AP (2010). Posterolateral transfibular approach to tibial plateau fractures: technique, results, and rationale. J Orthop Trauma.

[26] Thomas P, Richard E, Christopher G. AO Principles of fracture management, second expanded edition. Switzerland: AO Publishing; 2007.

[27] Sun H, Zhai QL, Xu YF, Wang YK, Luo CF, Zhang CQ (2015). Combined approaches for fixation of Schatzker type II tibial plateau fractures involving the posterolateral column: a prospective observational cohort study. Arch Orthop Trauma Surg.

[28] Sciadini MF, Sims SH (2013). Proximal tibial intra-articular osteotomy for treatment of complex Schatzker type IV tibial plateau fractures with lateral joint line impaction: description of surgical technique and report of nine cases. J Orthop Trauma.

[29] Carlson DA (1998). Bicondylar fracture of the posterior aspect of the tibial plateau. A case report and a modified operative approach. J Bone Joint Surg Am.

[30] Rasmussen PS (1973). Tibial condylar fractures. J Bone Joint Surg Am.

